# Exploring Cardiac Sympathetic Denervation in Transthyretin-Mediated Hereditary Amyloidosis (ATTRv): Insights from ^123^I-mIBG Scintigraphy

**DOI:** 10.3390/diagnostics15040508

**Published:** 2025-02-19

**Authors:** Maria Silvia De Feo, Chiara Cambieri, Eleonora Galosi, Viviana Frantellizzi, Cristina Chimenti, Marco Luigetti, Maria Ausilia Sciarrone, Francesca Graziani, Luca Leonardi, Beatrice Musumeci, Laura Libonati, Federica Moret, Edoardo D’Andrea, Matteo Di Giulio, Matteo Garibaldi, Francesca Forcina, Andrea Truini, Giuseppe De Vincentis, Maurizio Inghilleri, Marco Ceccanti

**Affiliations:** 1 Department of Radiological Sciences, Oncology and Anatomo Pathology, Sapienza University of Rome, 00151 Rome, Italy; mariasilvia.defeo@uniroma1.it (M.S.D.F.);; 2 Department of Human Neuroscience, Sapienza University of Rome, 00185 Rome, Italymarco.ceccanti@uniroma1.it (M.C.); 3 Department of Clinical, Anesthesiological and Cardiovascular Sciences, I School of Medicine, Sapienza University of Rome, 00185 Rome, Italy; 4 Dipartimento di Neuroscienze, Università Cattolica del Sacro Cuore, 00168 Rome, Italy; 5 Dipartimento di Neuroscienze, Organi di Senso e Torace, Fondazione Policlinico Universitario Agostino Gemelli IRCCS, 00168 Rome, Italy; 6 Department of Cardiovascular Medicine, Fondazione Policlinico Universitario Agostino Gemelli IRCCS, 00168 Rome, Italy; 7 Neuromuscular and Rare Disease Centre, Neurology Unit, Sant’Andrea Hospital, 00189 Rome, Italy; 8 Department of Clinical and Molecular Medicine, Sapienza University of Rome, 00185 Rome, Italy; 9 Department of Neurology, Mental Health and Sensory Organs (NESMOS), Sapienza University of Rome, 00185 Rome, Italy; 10 IRCCS Neuromed, 86077 Pozzilli, Italy

**Keywords:** ATTRv, amyloidosis, transthyretin, heart denervation, ^123^I-mIBG scintigraphy

## Abstract

**Background/Objectives:** Hereditary transthyretin-mediated amyloidosis (ATTRv) is a rare disease characterized by the deposition of amyloid in the heart and peripheral nerves, particularly affecting small fibers. This study aims to evaluate autonomic cardiac involvement in ATTRv. **Methods:** Twelve patients with ATTRv and twelve sex- and age-matched healthy subjects underwent ^123^I-mIBG scintigraphy to evaluate the early and late heart-to-mediastinum ratio (eH/M and lH/M), ^99m^Tc-HDP bone scan scintigraphy, and neurophysiological assessments. Data were analyzed in relation to functional cardiac and neurologic scales (NYHA and FAP scales). **Results:** Patients with ATTRv exhibited significant cardiac denervation, as demonstrated by the reduction in early and late H/M ratios compared to the control group (eH/M: 1.48 ± 0.08 vs. 1.89 ± 0.05, *p* < 0.001; lH/M: 1.39 ± 0.08 vs. 2.01 ± 0.05, *p* < 0.001). Values of eH/M and lH/M < 1.6 effectively differentiated patients with ATTRv from the healthy controls. Cardiac denervation correlated with interventricular septal thickness and the Perugini score but was not related to neurophysiological assessments or NYHA and FAP scales. **Conclusions:** Ultimately, ^123^I-mIBG scintigraphy is an effective tool for assessing cardiac denervation in patients with ATTRv.

## 1. Introduction

Hereditary transthyretin amyloidosis (ATTRv, “v” for variant) is a disease caused by the systemic deposition of mutant transthyretin (TTR). It is classified as a rare disease, with a prevalence in Italy of 4,33/million [1], although some regions show higher prevalence rates [2,3]. The heart and nervous systems, particularly the peripheral nerves, are the most commonly involved tissues, though other tissues may also be affected [4,5,6].

Amyloid deposition in the heart is detectable using bone tracer scintigraphy, which is recognized as a non-invasive diagnostic method due to the high affinity of TTR amyloid for bone tracers [7]. The impact of amyloid deposition extends beyond mere steric hindrance. Numerous studies have demonstrated its direct toxic effects [8], including pro-inflammatory gene activation [9], mitochondrial dysfunction, impaired antioxidant defense mechanisms [10], and oxidative stress induction in cardiomyocytes [11].

The natural course of the disease is dire. Death typically ensues within a few years after symptom onset, primarily due to cardiac complications. Quality of life is severely affected by neurological disability, which may progress to wheelchair dependence or complete immobility. Additionally, quality of life is worsened by autonomic symptoms, including orthostatic hypotension, gastrointestinal dysmotility, altered sweating and salivation, and, in men, erectile dysfunction.

In the context of the peripheral nervous system, patients primarily exhibit dysfunction in small sensory fibers, particularly the autonomic nervous system [2,12], especially in endemic countries. In non-endemic countries, small-fiber dysfunction is often associated with damage to large sensory and motor fibers [2]. In both cases, small-fiber impairment leads to specific symptoms related to neuropathic pain and dysautonomia [13,14].

Different tools can assess small-fiber dysfunction. In an appropriate clinical context, particularly in ATTRv, skin biopsy is considered the gold standard for diagnosing small-fiber neuropathy [15,16]. This procedure involves the histological analysis of intraepidermal small nerve fibers, which are primarily responsible for thermodolorific sensations. In some cases, the innervation of skin appendages can also be assessed [16]. Psychometric and functional tests, such as quantitative sensory testing and Sudoscan, are frequently used to assess small fibers in ATTRv [15,17,18]. Recently, neurophysiological assessments of silent periods have emerged as an additional diagnostic approach [12].

The autonomic nervous system, through the sympathetic and parasympathetic divisions, innervates almost all organs, modulating their activity. The heart is also autonomically innervated, with parasympathetic fibers decreasing the heart rate (chronotropy) and sympathetic fibers increasing it.

Scintigraphy with the ^123^I-Metaiodobenzylguanidine (^123^I-mIBG) tracer is a diagnostic tool that assesses the sympathetic innervation of the left ventricle, using a radiolabeled analog of norepinephrine, which shares uptake and storage mechanisms with norepinephrine in adrenergic nerve terminals [19].

The most evaluated parameter is the ratio between heart and mediastinum uptake at early and late imaging time points. Early H/M (eH/M) reflects ^123^I-mIBG influx into extraneural spaces, while late H/M (lH/M) reveals neuronal uptake and correlates with sympathetic nerve terminal function and activity [20].

Few studies have utilized this tool in ATTRv [21,22,23,24,25,26]. This study aimed to characterize the patterns of myocardial scintigraphy with ^123^I-mIBG uptake in a sample of patients with ATTRv, correlating the findings with clinical and neurophysiological characteristics and bone tracer uptake.

## 2. Materials and Methods

Twelve patients diagnosed with ATTRv at amyloidosis referral centers in Rome (Policlinico Umberto I, Policlinico Agostino Gemelli, and S. Andrea Hospital) underwent ^123^I-mIBG scintigraphy within six months of a prior myocardial bone tracer scan (^99m^Tc-HDP).

Demographic data, genetic data, interventricular septal thickness (measured via ultrasound), and Perugini score on ^99m^Tc-HDP bone scan scintigraphy were collected for each patient. The Perugini score [27] is a semi-quantitative assessment of cardiac radiotracer uptake using ^99m^Tc-HDP. In particular, grade 0 means no cardiac uptake, grade 1 means cardiac uptake that is less than the rib uptake, grade 2 means cardiac uptake similar to the rib uptake, and grade 3 means cardiac uptake greater than the rib uptake, with a mild or absent rib uptake. Additionally, the NYHA (New York Heart Association) functional classification for heart failure severity and the FAP (familial amyloid polyneuropathy) stage were evaluated, along with sensory action potential (SAP) amplitudes and compound muscle action potential (cMAP) amplitudes.

The FAP stage is a neurological functional classification dividing patients as being asymptomatic (FAP0), having sensory neuropathy (FAP1), requiring assistance for walking (FAP2), or being bedridden or wheelchair-bound (FAP3) [28].

Twelve age- and sex-matched healthy subjects were included as the controls. These individuals were selected from a historical dataset of patients evaluated for suspected Parkinson’s disease in whom the diagnosis had not been confirmed.

### 2.1. ^99m^Tc-HDP Bone Scan Scintigraphy

Patients received an intravenous injection of 740 MBq of 99mTc-HPD within six months of ^123^I-mIBG scintigraphy. A dual-headed gamma camera equipped with a low-energy high-resolution (LEHR) collimator (Infinia, GE Healthcare, Milwaukee, WI, USA) and a photopeak energy centered at 140 keV (±10%) was used for image acquisition two hours after tracer injection. A planar image of the thorax (256 × 256 matrix, zoom 1) was acquired, followed by a myocardial single-photon emission computed tomography (SPECT) study. The planar image was analyzed, and cardiac uptake was assessed both qualitatively through the Perugini visual score and semi-quantitatively by calculating the heart-to-contralateral ratio (H/CL ratio).

### 2.2. Neurophysiological Assessments

Electrophysiological studies were performed using a Micromed Myoquick EMG machine 1400 (Micromed System Plus Evolution 1.04.0097 S.p.A., Treviso, Italy). The SAP peak-to-peak amplitude was recorded from the sural and ulnar nerves on the left side; the cMAP baseline-to-peak amplitude was recorded using Ag/AgCl surface electrodes (Disposable Adhesive Surface Electrodes, Spes Medica S.r.l., Genoa, Italy) on the left abductor digiti minimi (ADM), with supramaximal electrical stimulation of the ulnar nerve.

### 2.3. ^123^I-mIBG Scintigraphy

A gradual intravenous injection of ^123^I-mIBG (111–185 MBq) was used for scintigraphic imaging. One hour before its injection, a 400 mg dose of potassium perchlorate or potassium iodide oral solution (Lugol’s solution) was administered orally. A dual-head gamma camera (Infinia, GE Healthcare, Milwaukee, WI, USA) with LEHR parallel-hole collimators and a photopeak energy centered at 159 keV (±10%) was used for image acquisition 15 min (early phase) and 4 h (delayed phase) after tracer injection. Planar images were acquired over ten minutes with a 128 × 128 matrix, zoom 1. A 7 × 7 pixel region of interest (ROI) was placed on the mediastinum at the midline of the upper chest, and another ROI was placed on the left ventricle. The early and delayed heart-to-mediastinum ratios (eH/M and lH/M) were calculated by dividing the mean count per pixel in the myocardium by that in the mediastinum.

### 2.4. Statistical Analysis

Demographic and clinical data were expressed as mean ± standard error of the mean (SEM). Continuous variables were compared using Student’s *t*-test or the Mann–Whitney U-test/Kruskal–Wallis test, depending on data distribution, which was assessed through the Shapiro–Wilk test.

Curve estimation was performed to evaluate associations between variables, particularly to investigate the relationship between ^123^I-mIBG scintigraphic parameters, interventricular septal thickness, and neurophysiological data. A receiver operating characteristic (ROC) curve analysis was conducted to establish cut-off values for distinguishing patients with ATTRv from the healthy controls. The optimal threshold was determined using Youden’s index.

All statistical analyses were performed using IBM SPSS Statistics version 27.0; statistical significance was set at *p*  <  0.05.

## 3. Results

### 3.1. Demographic Data

The age and gender of the patients with ATTRv were not significantly different compared to healthy subjects. Val50Met and Non-Val50Met mutations were balanced. Echocardiographic parameters and cardiac and neurological functional scales are reported in Table 1.

### 3.2. ^99m^Tc-HDP Bone Scan Scintigraphy

**Figure 1:**
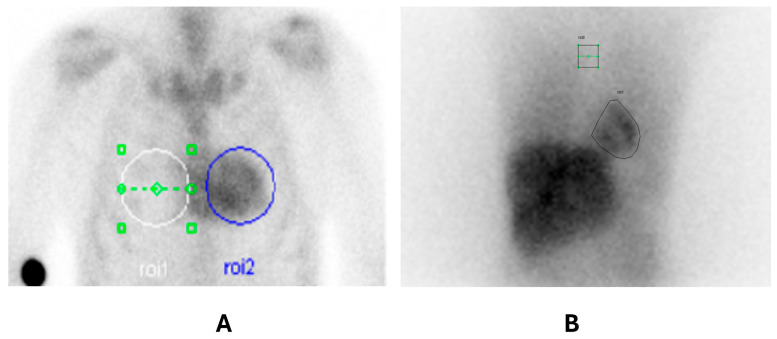
(**A**) ^99m^Tc-HDP planar chest image. Application of the semi-quantitative method with the calculation of the heart-to-contralateral ratio (H/CL ratio): the circle of the region of interest being studied is in blue (ROI 2), and the contralateral comparison circle is shown in white (ROI 1). (**B**) ^123^I-mIBG planar anterior chest image of a patient with lH/M:1.88. A manually drawn ROI is placed on the heart (ROI 1) and a fixed ROI is placed in the mediastinum (ROI 0).

**Figure 2:**
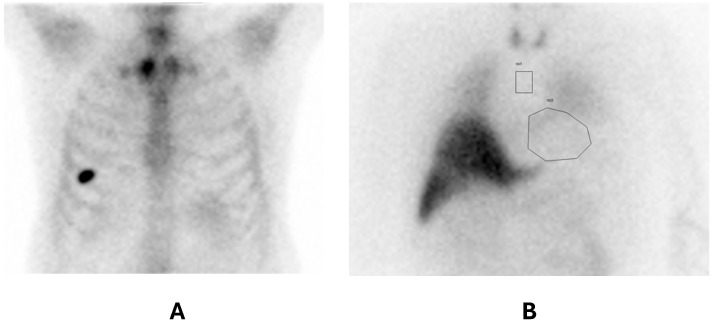
(**A**) ^99m^Tc-HDP planar chest image of the patient with a Perugini score of 0 and a Phe84Leu mutation. (**B**) ^123^I-mIBG planar anterior chest image of the patient with the Phe84Leu mutation. lH/M:1.31. A manually drawn ROI is placed on the heart (ROI 0) and a fixed ROI is placed in the mediastinum (ROI 1).

The Perugini score on ^99m^Tc-HDP bone scan scintigraphy was 0 in one patient, 2 in two patients, and 3 in nine patients (Figure 1A,B). The patient with a Perugini score of 0 had a Phe84Leu mutation, previously described as a mutation which typically does not show bone tracer uptake (Figure 2A).

### 3.3. Neurophysiological Assessments

The mean SAP amplitude values from sural and ulnar nerves in patients with ATTRv were 4.2 ± 2.1 µV and 4.2 ± 1.2 µV, respectively, with only one patient in whom the SAP from the sural nerve was not recordable. The mean cMAP amplitude from the ulnar nerve in patients with ATTRv was 7.7 ± 1.3 mV.

### 3.4. ^123^I-mIBG Scintigraphy

**Figure 3:**
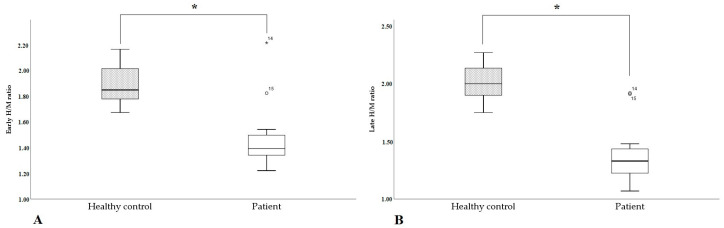
(**A**). Early H/M ratio in healthy controls and patients with ATTRv. (**B**). Late H/M ratio in healthy controls and patients with ATTRv. * *p* < 0.05. The boxes indicate the upper and lower quartiles, and the whiskers indicate the minimum to the maximum value.

^123^I-mIBG scintigraphy showed a significant reduction in both early and late H/M ratios in patients with ATTRv compared to the control group (eH/M: 1.48 ± 0.08 vs. 1.89 ± 0.05, *p* < 0.001; lH/M: 1.39 ± 0.08 vs. 2.01 ± 0.05, *p* < 0.001; Figure 3A,B). An ROC curve estimation demonstrated 1.6 as a good diagnostic value for both early and late H/M in discerning healthy subjects from patients with ATTRv (eH/M: 1.605: sensitivity 100%, specificity 93.3%, AUC 0.885, *p* < 0.01; lH/M: 1.615: sensitivity 100%, specificity 93.3%, AUC 0.955, *p* < 0.001).

Both early and late H/M ratios were directly related to interventricular septal thickness (eH/M: R^2^ 0.339, *p* < 0.05; lH/M: R^2^ 0.391, *p* < 0.05) but unrelated to NYHA or FAP functional classes, neurophysiological assessments, and the presence of Val50Met or non-Val50Met mutations.

**Figure 4:**
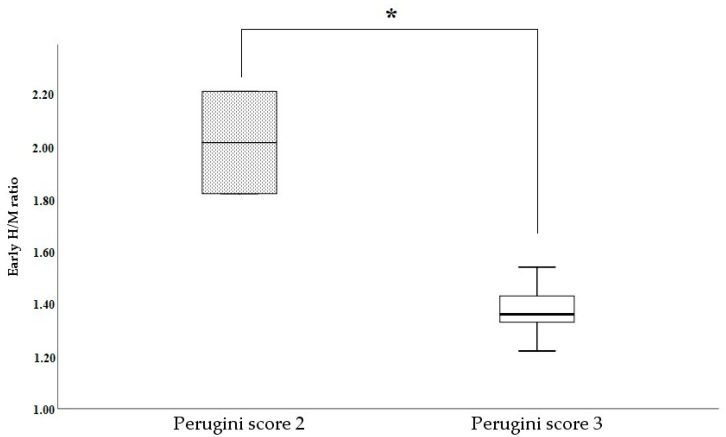
Early H/M ratio in patients with Perugini scores 2 and 3. * *p* < 0.05. The boxes indicate the upper and lower quartiles, and the whiskers indicate the minimum to the maximum value.

Patients with Perugini grade 3 showed reduced heart innervation compared to those with grade 2 (eH/M: 1.37 ± 0.03 vs. 2.01 ± 0.19, *p* < 0.05; lH/M: 1.29 ± 0.04 vs. 1.91 ± 0.01, *p* < 0.05; Figure 4). The only patient with ATTRv with a Perugini score of 0 and a Phe84Leu non-uptake mutation had an early and late H/M of 1.38 and 1.31 (Figure 2A,B), respectively, demonstrating significant denervation despite normal ^99m^Tc-HDP bone scan scintigraphy. This patient also exhibited an increased interventricular septal thickness (16 mm).

## 4. Discussion

Like any organ, the heart is innervated by the autonomic nervous system via ortho-sympathetic and parasympathetic small fibers, which modulate its specific activity and function. ^123^I-mIBG scintigraphy is considered the gold standard for the ortho-sympathetic characterization of cardiac innervation.

Sympathetic cardiac innervation originates from the intermediolateral cell column of the (C8)-T1-T4-(T5) spinal cord segments [29]. Nerve fibers exit the spinal cord via anterior roots, join the spinal nerves, and pass through the anterior ramus and white ramus communicants to reach the paravertebral ganglia of the sympathetic chain. These ganglia include the superior, middle, and vertebral cervical ganglia, as well as the stellate ganglion (formed by the fusion of the inferior cervical and first thoracic ganglia). Here, the fibers synapse with postganglionic neurons, which extend to the heart via cardiac nerves. Cardiac sympathetic nerves enter the heart at the vascular pole and form three main branches that follow the coronary arteries [30]: the left coronary cardiac nerve (along the anterior interventricular branch of the left coronary artery), the left lateral cardiac nerve (along the circumflex artery), and the right coronary cardiac nerve (along the right coronary artery).

One of the primary pathophysiologic anomalies linked to heart failure is the activation of the sympathetic nervous system. Heart rate acceleration (positive chronotropic effect) and an increase in myocardial contractility (positive inotropic effect) are two key cardiovascular functions of cardiac sympathetic innervation [31]. Myocardial imaging with ^123^I-mIBG is useful for detecting abnormalities in the myocardial adrenergic nervous system in patients with chronic heart failure (CHF), has significant prognostic value, and helps predict sudden death [32,33].

In the multicenter ADMIRE-HF (ADreView Myocardial Imaging for Risk Evaluation in Heart Failure) study, which prospectively evaluated the prognostic significance of cardiac ^123^I-mIBG imaging in 961 stable patients with CHF with NYHA class II or III and an LVEF ≤ 35%, ^123^I-mIBG was shown to be a predictor of cardiac events, including heart failure (HF) progression, fatal arrhythmia, and cardiac death [34]. Cardiac mortality, fatal ventricular tachycardia (VT), or HF progression were all predicted by a predetermined H/M cut-off ratio of 1.6. With a 2-year event rate of 37% versus 15% (*p* < 0.001), individuals with H/M < 1.6 had a considerably greater risk of a cardiac event than patients with H/M > 1.6.

In addition to acting as a useful dichotomous predictor of prognosis, H/M has prognostic implications over the whole range of outcome values for all event categories, except ventricular arrhythmias, according to a meta-analysis of six trials involving 636 patients with CHF [35]. Even in studies emphasizing gender differences, the heart/mediastinum ratio remains clinically relevant. In a study of 306 patients, ^123^I-mIBG was found to be a more effective tool for predicting adverse events in male patients than in female patients [36].

From a cardiac pathology perspective, several studies have demonstrated that cardiac resynchronization therapy (CRT) improves cardiac sympathetic activity as assessed by ^123^I-mIBG [37,38]. Early and late H/M ratios were independent predictors of response to CRT when improvement in the LVEF was utilized as a response parameter, according to a multicenter trial involving 78 patients with stable CHF. The late H/M ratio was an independent predictor of LVEF improvement to >35% (*p* = 0.0149), and the early H/M ratio was an independent predictor for LVEF improvement >10%. To optimize patient selection for CRT, ^123^I-mIBG cardiac scintigraphy serves as an excellent tool [35].

Furthermore, although ^123^I-mIBG scintigraphy is associated with arrhythmic events in patients with CHF with an implantable cardioverter–defibrillator (ICD) for primary and secondary prevention, no association was found between the ^123^I-mIBG scan-derived parameters and appropriate ICD therapy [39,40].

^123^I-mIBG, as a nonspecific amyloid radiotracer, has also been studied in cardiac amyloidosis. Damage to the myocardium’s sympathetic innervation may occur before left ventricular hypertrophy or clinically apparent heart disease develops, according to a study conducted with ^123^I-mIBG imaging in patients with a familial form of amyloidosis (ATTRv Val50Met) [41]. In a different study, the severity of polyneuropathy, measured through a functional scale, correlated with decreased lH/M [42].

Coutinho et al. demonstrated that ^123^I-mIBG imaging is a valuable prognostic indicator for ATTRv. Individuals with late H/M < 1.60 appeared to benefit from liver transplantation and were more likely to have an adverse outcome [25].

In this study, cardiac ortho-sympathetic denervation was demonstrated by the reduction in early and late H/M ratios in patients with ATTRv compared to age- and sex-matched healthy controls. A cut-off value of 1.6 for both eH/M and lH/M could discriminate between these two groups with high sensitivity and specificity. This value is particularly intriguing, as patients with HF were demonstrated to be at risk of all-cause and cardiac death, arrhythmic events, sudden cardiac death, or potentially life-threatening arrhythmias when the lH/M was <1.6 [43].

Patients with ATTRv, even in this sample, showed cardiomyopathy with heart failure and preserved ejection fraction (HFpEF). The majority of patients with ATTRv in this study had lH/M (and eH/M) below the 1.6 threshold, which was also demonstrated to be the best cut-off value to distinguish them from the healthy controls. An increase in the arrhythmic burden has been described in ATTRv [43,44,45,46], and its reduction has been considered in some post hoc analyses of disease-modifying drugs [47]. Heart denervation could affect mortality and arrhythmic burden in patients with ATTRv, and ^123^I-mIBG scintigraphy could identify patients at the highest risk.

Heart denervation seems to proceed in parallel with cardiomyopathy, although an earlier involvement cannot be ruled out. In fact, eH/M and lH/M are related to interventricular septal thickness and the Perugini score. A previous study demonstrated that positive ^123^I-mIBG scintigraphy was associated with a higher risk of positive DPD scintigraphy in carriers of late-onset Val50Met and other variants [26].

Interestingly, the only patients with Phe84Leu included in this study exhibited significant denervation, despite a Perugini score of 0 (Figure 2A,B). Bone scintigraphy has a low sensitivity for detecting amyloid deposits in the Phe84Leu mutation [48], probably due to a reduced affinity to the bone tracer. The Phe84Leu mutation has been associated with moderate dysautonomia. Mazzeo et al. [49] described 28 patients with this mutation, reporting dysautonomic symptoms, such as orthostatic hypotension, diarrhea/constipation, erectile dysfunction, urinary incontinence, or xerostomia, occurring within four years of disease onset.

Our patient with Phe84Leu had an increased interventricular septal thickness (16 mm) and significant denervation (eH/M 1.38, lH/M 1.31), suggesting that heart denervation and infiltration proceeded in parallel in this patient, independently of the ability of DPD scintigraphy to detect amyloid deposition. ^123^I-mIBG scintigraphy could be used to demonstrate heart involvement in this specific mutation, but further studies are necessary to confirm these findings.

On the contrary, denervation was unrelated to the cardiologic and neurologic functional scores (NYHA and FAP classes). Even the neurophysiological assessments were not related to heart denervation. In this regard, we emphasize that standard neurophysiologic assessments only consider myelinated high-diameter fibers, structurally different from the unmyelinated small fibers of the autonomic nervous system. This could justify the lack of a relationship between functional scores, neurophysiology, and ^123^I-mIBG scintigraphy.

The small sample represents a major limitation of this study. Further studies also involving asymptomatic ATTRv carriers are necessary to confirm these data, including subgroup analyses of different mutations. Moreover, imaging should be compared to electrocardiographic data, and a longitudinal follow-up could identify a predictive role of ^123^I-mIBG scintigraphy in detecting life-threatening events.

Finally, treatments have become available for ATTRv. In particular, TTR stabilizers and RNA interference drugs have been shown to slow the progression of ATTRv, reducing major cardiac events, delaying functional decline, and extending life expectancy [50,51,52,53]. In drug approval studies, autonomic function has been considered only as an exploratory endpoint. Other drugs, such as CRISPR-Cas9 [54,55] and monoclonal antibodies [56], are expected to further improve ATTRv therapy through a gene-silencing mechanism or amyloid burden removal. The use of ^123^I-mIBG could provide further insights into the effects of these treatments on cardiac innervation.

## 5. Conclusions

This exploratory study suggests a role for ^123^I-mIBG scintigraphy in patients with ATTRv. The patients with ATTRv in our study appeared to be significantly denervated compared to their sex- and age-matched controls. Considering that heart denervation could increase their risk of malignant arrhythmias and sudden death, patients with ATTRv could undergo ^123^I-mIBG for risk stratification. Specific mutations, namely Phe84Leu, could be examined with ^123^I-mIBG to improve disease management.

## Figures and Tables

**Table 1:** Demographic data. “N/A”: not applicable.

	Patients with ATTRv (*n* = 12)	Control Group (*n* = 12)
Age (years, mean ± SD)	68.3 ± 2.4	68.1 ± 2.3
Sex (M/F)	11/1	11/1
Mutation Type	Val50Met (*n* = 6), Non-Val50Met (*n* = 6)	N/A
NYHA Class	I (*n* = 3), II (*n* = 8), III (*n* = 1)	I (*n* = 12)
FAP Stage	1 (*n* = 4), 2 (*n* = 6), 3 (*n* = 2)	N/A
Interventricular Septal Thickness (mm)	17.5 ± 1.1	N/A
Ejection Fraction (EF)	42.2 ± 4.1	N/A
Perugini Score	0 (*n* = 1), 2 (*n* = 2), 3 (*n* = 9)	N/A
Early H/M Ratio (eH/M)	1.48 ± 0.08	1.89 ± 0.05
Late H/M Ratio (lH/M)	1.39 ± 0.08	2.01 ± 0.05

## Data Availability

Data will be shared by the corresponding author upon reasonable request.
